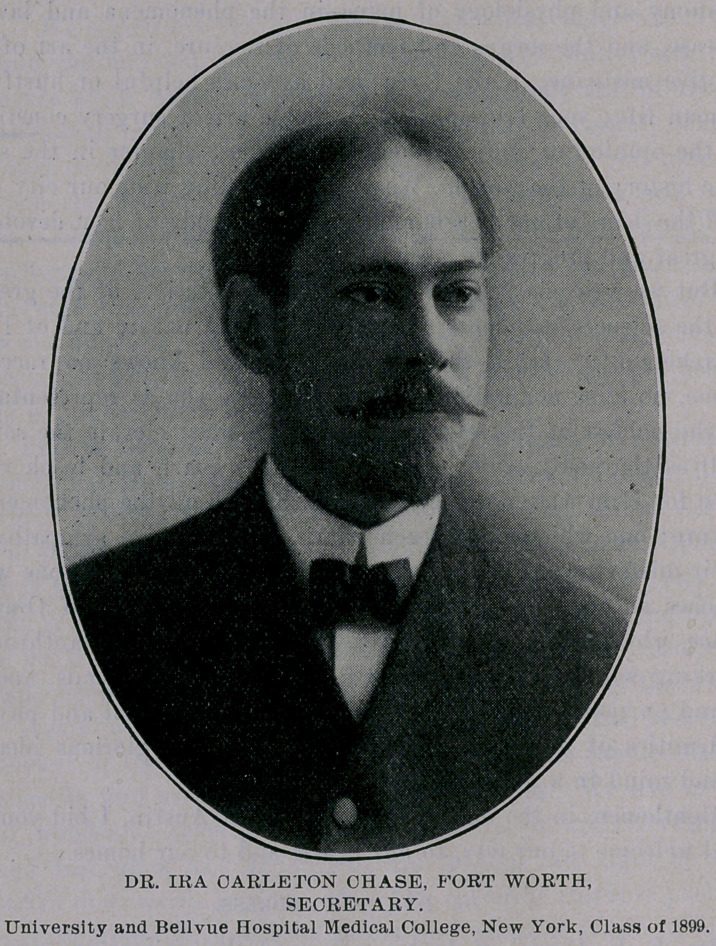# State Medical Association of Texas

**Published:** 1904-05

**Authors:** 


### TEXAS MEDICAL JOURNAL.

AUSTIN, TEXAS.

A MONTHLY JOURNAL OF MEDICINE AND SURGERY.

EDITED AND PUBLISHED BY
F. E. DANIEL, M. D.
Mrs. F. E. DANIEL, Managing Editor.

ASSOCIATE EDITOR:
WITTEN BOOTH RUSS, M. D., San Antonio, Texas.

Published Monthly at Austin, Texas. Subscription price $1.00 a year in advance.
Eastern Representative: John Guy Monihan, St. Paul Building, 220 Broadway, New York City.
Official organ of the West Texas Medical Association, the Houston District Medical Association, the Austin District Medical Society, the Brazos Valley Medical Association, the Galveston County Medical Society, and several others.

### STATE MEDICAL ASSOCIATION OF TEXAS.

**The Big Meeting at Austin.**

The thirty-sixth annual meeting of the State Medical Association of Texas was held at Austin as per announcement, April 25, 26, 27, 28, 29, ult. There was a very large attendance and the utmost enthusiasm and harmony prevailed. It broke the record for numbers, and all went away saying they never had so good a time in their lives. Members who had dropped out years ago and who had not attended a meeting since the woods were burnt, put in appearance, and caught on with renewed interest. Lots of new faces turned up also, and it was a delight to meet and shake hands with hundreds of recruits. It was doubly pleasant to feel the cordial grip of the old, tried and true,—the old guard I call them. The Houston doctors who have charge of the arrangements for the 1905 meeting, are on their mettle. The spread here has pricked the side of their intent—or something like that—and they say that next April they will lay it over Austin in the way of a rousing meeting and entertainments if it takes $5000 to do it, and they'll get the money.

I do not think it would particularly interest anybody to give the titles of papers read, and as I have not room to do it or publish the discussions, I just cut it out, and give below a sort of running

F. E. DANIEL, M. D.,

President State Medical Association of Texas, 1904–1905.

Virginia, 1839; M. D. from New Orleans School of Medicine, February 1862; Surgeon Confederate States Army, 1862-1865; First Vice-President American International Congress on Tuberculosis, St. Louis, 1904; Editor Texas Medical Journal, 1885 to 1904; etc.

account of the meeting, together with some of the pretty speeches. The House of Delegates met and organized Monday, April 25th.

TUESDAY, APRIL 26.

The first session of the State Medical Association met at 10 o'clock in the auditorium of the University of Texas and the meeting was well attended. The convention was called to order by Dr. T. J. Bennett, chairman of the committee of arrangements. He declared the convention formally opened.

Dr. H. S. Werlein, pastor of the Tenth Street Methodist church, invoked divine blessing upon the members of the association, after which Mayor White officially welcomed the association to Austin on the part of the city. He declared that Austin had been turned over to them and everything therein was theirs. He turned over the keys of the city to the doctors. His speech was short but very appropriate to the occasion.

DR. BRIGGS'S ADDRESS OF WELCOME.

Rev. R. J. Briggs, M. D., welcomed the association on behalf of the citizens of Austin. He said:

Gentlemen of the State Medical Association.—I have the honor and pleasurue of bidding you welcome today in the name of the citizens of Austin. I wish to assure you at the outset that our welcome is not a mere conventionalism arising out of the courtesies of a polite and elegant society. It means far more than this. Although extended thus publicly, and to you collectively, our welcome is as cordial and sincere as that which you individually meet at the doors of our homes, when you enter them as the angels of hope, of health, and of healing.

It is as the truest friends of the family that we welcome you first of all today. It is the sacred and tender relation of the family physician which lies back of and glorifies this scene in the eyes of our citizens today. No man stands nearer to our hearts; no man is more honored in our souls; no man is more truly loved and implicitly trusted than the family physician. And so, speaking for the citizens of Austin today, I but voice in the form of public welcome the love and confidence by which you are linked in closest bonds to the thousands of homes that dot the surface of our fair State.

Again we welcome you as representatives of the world's truest philanthropy. We live in a sorely tormented world. No part of it is undarkened by sorrow, undesolated by death. Early and late, from populous city and sequestered hamlet, from the palaces of the rich and the hovels of the poor, there comes that never ceasing cry of agony which weeps by the bedside of the sick and wails above the forms of the dying and the dead. In a world where such dreadful experiences are so constant and so universal, who can doubt that in medicine philanthropy finds its tenderest and most comprehensive mission.

We sometimes feel pity for the man whose whole life is passed amid the maimed, the halt and diseased, who always sees human nature at its worst. The spirit of philanthropy and of reverence for human nature is in no man so constantly and sorely taxed and tested as in the physician. For in the sick room you most often reach the bottom of man's meanness, his selfishness and cowardice. There you see human nature in decay; as unbeautiful as the workings of continual weakness, pain and want can make it. And the man who, unrepelled by all this, crosses the threshhold gladly as the angel of true service, with heart too full and hand too busy for aught but the gracious ministries of helpfulness and healing—he is a true philanthropist.

This is the spirit which glorifies the practice of medicine and which has flowered out in its history into many a blossom like that which glorified the passion tree which stood on Mount Calvary—the passion tree whereon self is crucified for others. Gentlemen, it is the aroma of that spirit which you bring into our city today; and it falls upon the spiritual senses of our people as the fragrance of an unseen bank of violets and makes all the air redolent, and falls upon and charms the senses of every passer by the way.

Again we welcome you as representatives of the greatest and noblest of the sciences. It is the greatest in its intellectual excellence as well as in its moral disinterestedness. We live in an age of wonderful scientific activity. Discoveries are crowding thick and fast upon us. Inventions are leaping into light as meteors strike downwards into the air. The scientific spirit is pushing its investigations far and wide across the realms of truth. It is grappling with, subverting and subduing all the obstacles of earth and time and space—climbing from crest to crest of marvelous achievement, until all things material are trodden under foot or enlisted in its service. And foremost in this shining march of progress moves the science of medicine. It is amazing to contemplate the progress and triumphs of medical science during the last fifty years. Endowed with marvelous vitality, laying all nature and art under contribution to its progress, commanding the genius and devotion of thousands of master minds, its triumphs have been marked by immeasurable splendor. Quickened by exhaustless energies which halt only where the finite ends and the infinite begins, it has moved down the path of the centuries exploding the errors of the past, dissipating the obscurities of the present and illuminating the sky of the future with the bright promise of still grander triumphs to come. Your researches and discoveries in pathology—the morbid anatomy and physiology of man—in the phenomena and laws of disease, and the means and methods of its cure, in the art of preventive medicine, in the forces and agencies helpful or hurtful to human life; your triumphs in the noble art of surgery constitute, in the opinion of your speaker, the proudest chapter in the scientific history of the world. We are glad to fling wide our city gates and the doors of our hearts and homes to a body of men devoted to so great and progressive a science.

But we welcome you not alone as representatives of the greatest of the sciences—students of the mysteries of nature and of life—searchers after truth that is universal, and knows no race nor clime, no tribe nor nation—but we welcome you as representatives of the noblest of the sciences—one which must elevate the soul as well as the body; which must chasten the heart, and teach reverence for Him who moves behind and through all the phenomena of nature; one which must teach charity for all men, sympathy for their misfortunes and patience with their transgressions; one which teaches us our own littleness and the awful majesty of Omnipotence, which makes wiser, braver, better men, true philanthropists, working with lofty aim toward definite, benevolent ends, and the grand purpose of which is the relief of all the mental and physical infirmities of mankind—the attainment of that glorious ideal—a sound mind in a sound body.

Gentlemen, in the name of the citizens of Austin, I bid you cordial welcome to our city, to our hearts and to our homes.

### DR. DANIEL'S ADDRESS.

Dr. F. E. Daniel of Austin welcomed the visitors on behalf of the medical profession. He said:

Brothers and colleagues, I greet you! To each of you individually I extend the glad hand and bid you welcome. On behalf of Travis County Medical Society, whose honored guests you are, I welcome you to the queenly capital of the proudest State in America. When, in that ancient day, to be a Roman was greater than a king, Rome, from her seven hills, ruled the world. Austin, crowning, like a diadem, the beautiful cedar clad hills of the Colorado, from her throne of beauty rules an empire greater than Rome. She guides the course and shapes the destiny of the great empire of the West, the future home of teeming millions. Here her laws are made. Here resides the authority for their enforcement and execution. Here sit her learned tribunals for their interpretation and construction, and here is located the pride of Texas,—her great

DR. IRA CARLETON CHASE, FORT WORTH,

SECRETARY.

University and Bellvue Hospital Medical College, New York, Class of 1899.

university. In this hotbed of learning there are ever incubating the embryo statesmen, philosophers, educators, authors, and scientists of the future, the coming doctors to take your places as units in that great and growing body, the medical profession. It is interesting to note the potentiality of the small boy. It is more so to contemplate the capacity for evolution and development of the embryo doctor. As the tiny seed sends out the tender sprout, whence springs the sturdy stalk, and the leaves unfold to kiss the summer sun and drink the dews of heaven, it completes its lifework with a burst of beautiful bloom and perfume to delight the senses, so may the Pasteur of the future be now germinating in the embryo student of medicine, or the Virchow or Helmholtz, the Herbert Spencer or Huxley, the Crooke or Roentgen, or that great American physician, the immortal Finlay, who has so recently given us the key to the problem of epidemic yellow fever. So mote it be.

The eyes of the world are upon Texas, and her medical men are in focus. Everywhere Texas physicians are esteemed the peers of the ablest in the land and their scientific work as given to the world in your yearly transactions receives the highest commendation of the medical press of the world. The State is proud of you. It is especially gratifying to see such an attendance, doubtless in response to the stimulus of reorganization and reawakened interest.

It is cause for congratulation to see the fruition of our long cherished hopes, a united, harmonious and prosperous medical profession.

I welcome you to a brief rest from your routine and exhausting labors. I know what they are. I know the wearing, anxious care, the fatigue, the lonesome night vigils by the bedside, the dreary, monotonous rides by night and day, through sun and sleet and snow and storm, without a thought of self, bent solely on your noble mission, happy and content if you can snatch from the jaws of death one curly-headed mother's darling or bring both mother and babe safely through the perils of parturition. I know what a joy and delight it is to lay down your burden even for a brief period and get away from the carking cares of your daily routine, and I welcome you to that rest. Here in the delight of scientific debate, the intellect is sharpened to keenest edge. You grasp the hand of the new found friend and greet the old, true and tried, from every section of this great big State. You meet their wives and sisters and daughters and enjoy their social intercourse. You meet the flower of womanhood of this, your capital city, and partake of their hospitality and the many amenities of social life, which, like flowers by the roadside, make less rugged your pathway through life.

While we have great cause for congratulation and rejoicing, we have, alas, cause for sorrow. In the brief year now sped into the past, beyond recall, a brief twelvemonth since last we met, death has extinguished the fires of life that but so lately burned brightly in the bosom of some of our dearest and best. We miss them and mourn them. Who that was at San Antonio last April does not recall the joyous, the winsome West, and see his face, radiant with happiness, and beaming with zeal and intelligence, and feel again the grasp of his honest hand in congratulation as he saw the work accomplished, so ardently desired, so earnestly sought. Around the secretary's chair will ever cluster the fondest recollections and the tenderest regrets for brave Hamilton West. It is a pleasing thought that they are with us in spirit today, that from the realms of yon bright elysium of peace, sped on angels' wings, they waft us a benediction and breathe a prayer for the harmony and success of the association they loved so well. Reverently we drop a tear to their memory and sigh in sorrow for their loss, but we must press on. Men perish, but their thoughts and deeds live on forever. Science never stops. We must push onward and upward to the accomplishment of our allotted work, "still achieving, still pursuing," go on and on, "heart within the God overhead." * * *

Again I welcome you. We welcome you to the warmest place in our hearts, welcome you to the innermost sanctuary of our homes. We welcome you to our most generous hospitality. We are yours. The door is open; the lid is off. If you don't see what you want, ask for it. If you do, take it.

### PRESIDENT PRATHER'S ADDRESS OF WELCOME.

President W. L. Prather of the University of Texas welcomed the delegates on behalf of the University. He said:

Mr. President, Ladies and Gentlemen: I esteem it a great privilege to welcome this body of distinguished men and women to the University of Texas and to extend to them most cordial greetings in behalf of the regents, the faculty and the students of this institution. We greet you not as strangers, but as friends, as fellow citizens of a great commonwealth, which here in Texas, amid these surpassingly beautiful hills of the Colorado, located its capital and designated this elevated and historic spot as the site of its future university.

Here, after years of delay, is gradually being realized the cherished design of the people of the Republic and of the State, the establishment within Texas of an institution for the instruction of the youths of the land in the higher branches of learning, which shall be so endowed, supported and maintained as to confer upon the sons and daughters of the State, whether rich or poor, a thorough education. This was esteemed a means whereby might be secured the attachment of the younger men of the State to the interests, the institutions, the rights of the State, and the liberties of the people.

Here is one institution which was founded by the republic and the State of Texas that represents no one locality, no single class, no special sex or sect of our people. Born of the wisdom, baptized with the love and endowed with the wealth of our fathers, it stands for the unity of Texas. It is your university and mine; it is the house for which our fathers laid the foundation for the benefit of their children and we are not strangers to each other here.

Here upon this commanding eminence, overlooking your great granite capitol, were planted during the Civil war the guns of General Magruder for the protection of the old capitol. Here today we are planting sentiments of patriotism in the hearts of the youth of Texas, which, in the years to come, will prove a surer defense to yonder capitol than shot and shell from cannons of brass and steel. And allow me to say, in passing, that it is wiser to train the youth of the land for the higher, nobler and more lasting triumphs of peace which advance civilization than for the victories of war which involve the destruction of lives and property and degrade mankind.

Here were spent the crowning years of that great lawgiver of Texas, O. M. Roberts, the ripeness and richness of whose legal attainments were devoted to the organization of the law department of this institution in conjunction with Judge Robt. S. Gould, the learned jurist and Christian gentleman, both ex-chief justices of our supreme court. Here the last years of the pure and devoted life of Dr. Leslie Waggener were spent. These consecrated lives have left their impress upon this institution, and the aroma of their virtues, like precious incense, lingers in these halls. Here for twenty years has been lived the pure and blameless life of Mrs. Helen M. Kirby, dean of women, the sister of your own accomplished and knightly Dr. R. M. Swearingen, she who is as distinguished among women as her brother was among men. The late Dr. Ashbel Smith and Dr. Thos. D. Wooten, members of your honored profession, were respectively the first and second Presidents of the Board of Regents. Two of the members of our exceptionally able Medical Faculty, Drs. J. F. Y. Paine and J. W. McLaughlin, are ex-presidents of your State Association. There are, therefore, many ties which bind you to this institution. We welcome you as citizens and as members of a learned profession which is doubly dear to us. We rejoice in the fact that your sessions will be held in this auditorium, and that for the four days of your meeting you will have the opportunity of seeing us daily as we are engaged on our work. Our daily recitations, our lectures and our laboratories will be open to each and all of you, so that you may come in at any time, remain as long as you wish and depart when you desire without explanation and without fear of interfering with our work. In brief, ladies and gentlemen, we are yours and you are ours.

I will not say to you as the mayors of our towns sometimes say, who wish to emphasize their hospitality, that you may take anything in sight and ask for anything you do not see. All that is here is the property of the State, and, therefore, yours, but we wish you to leave with us your benediction and take away only delightful impressions and charming recollections of your visit.

President Paschal responded to the address in a very eloquent manner. They didn't get it down. Section work then began; the House of Delegates went to work.

### PRESIDENT PASCHAL'S ADDRESS AND THE ANNUAL ORATION.

### THE NIGHT SESSION.

At the night session the entire association was present and opened by music furnished by the band from the State Insane Asylum.

Dr. T. J. Bennett of Austin then introduced Dr. Paschal of San Antonio, President of the association, who delivered his annual address.

He said in part:

The constitution of our State association requires that the President shall deliver an address, and in obedience to this mandate I have the honor to acknowledge your presence and I must crave your kind indulgence while I perform this duty.

It is not my purpose to trace the origin of diseases which began, no doubt, when the mythical Pandora opened the casket and liberated the germs that have ever since afflicted the human race, and will forever continue to do so; but it is to say something of that well worn subject, but to us an ever interesting one, of doctors, and their relation to the public. In speaking of them it will not be of Esculapius, or Apollo, of Hippocrates or Galen, of Velsalve, of Harvey, Jenner, Sydenham, or other great men who have adorned our profession—it will be of those who are dearer to us—of the physicians of our own great State of Texas. They by their professional attainments, their struggles against hardships, their unswerving devotion to duty, and their achievements in benefiting the people of the commonwealth, are justly entitled to the highest praise, and to be ranked foremost amongst the greatest benefactors that this State has ever known.

In speaking of diseases it will be of how to prevent some of them from reaping untimely harvests, and others from entailing untold sufferings, interrupting commerce, producing consternation and bringing financial ruin in their wake. We must remember that life at best is but a short span, and to lengthen it we devote our best years to unselfish labor. The greatest physicians are those who prevent diseases and not those who assist nature in curing them.

The State Medical Association has met here today for the first time since adopting the plan of organization, approved by the American Medical Association. It is a source of pride and pleasure, to know that fifty-one years ago the State Medical Association of Texas was born in this city, Austin. The plan under which we are now operating is similar to the one of 1853, which provided that county and district association should be subordinate branches of the parent association, and that county societies should be chartered by the State association upon the recommendation of the councilors, exactly as required now. In proof of this I submit the charter granted in 1853 by the State Medical association to the Bexar County Medical Society.

Dr. Paschal then gave a brief history of the association from its inception on January 17, 1853, up to the present time, when it is in such a flourishing condition. He next touched on medical legislation in which he showed the great difficulty encountered in getting through any measure pertaining to the medical profession.

Dr. Paschal then took up the question of public health and quarantine and declared:

"Unquestionably the most important subjects that should receive legislative consideration, are those appertaining to public health, and quarantine as it stands today, is far from satisfactory. Every county in this State has the right to decree a quarantine against another county, without the leave or sanction of the Governor or State Health Officer, whenever in the opinion of the county commissioners and county health officers some infectious or contagious disease justifies such action. The county health authorities are superior under the law to the State Health Officer."

He talked of the Boards of Health. He said that a better law relative to public health and quarantine should supercede the present one. The other subjects touched on were: "Adjudging the

Insane," the "Eleemosynary Institutions," and concluded by giving the proper credit where it belongs to the State Medical Association.

In the course of his remarks he paid a compliment to Dr. Wallace of Waco, who was one of the reorganizers of the present association in 1869 and one of the original organizers of the first association in 1853.

Among those present was Dr. C. A. L. Reed of Cincinnati, chairman of the American Medical Association committee on legislation, who was presented to the association.

The band then played another selection, after which Dr. Bennett introduced Dr. H. A. Barr of Beaumont, orator of the association, who delivered the annual oration. He said that in contemplating the demands of propriety which confronts him many emotions have been experienced, some of them conflicting and some of them not so. He took as his subject, "The Physicians of the Past and Present and Something of what They Have Accomplished." His effort was a masterly effort. He closed by paying a glowing tribute to the charming ladies present at the meeting.

This concluded the meeting for the evening. The members then made an inspection of the library and different departments of the University.

Election of Officers.—The House of Delegates announced Friday morning the following officers for the year: President, F. E. Daniel, Austin; Vice-Presidents, J. T. Moore, Galveston; C. E. Cantrell, Greenville; W. W. Shropshire, Yoakum; Secretary, Ira C. Chase, Fort Worth; Treasurer, R. F. Miller (re-elected), Sherman.

A Mascot.—Dr. W. R. Blailock of McGregor in a ringing speech full of flowers of rhetoric and gems of eloquence proposed that the association adopt a mascot, and nominated Mrs. F. E. Daniel to be its first and only one. He was seconded by Prof. Paine and others in pretty speeches, and the wife of the President-elect was adopted by acclamation as the association's mascot. Mrs. Daniel was escorted to the rostrum by Drs. T. T. Jackson of San Antonio and G. B. Foscue of Waco. She made a very graceful acknowledgment, which captured the convention. We have the pleasure of presenting our readers with her portrait herewith.

The newly elected officers, President Daniel, Vice-Presidents Moore, Cantrell and Shropshire, Secretary Chase and Treasurer

Miller were all in turn presented to the House of Delegates and all made what seemed to be very acceptable speeches, all of which our reporter failed to get. Dr. Daniel in presenting the Vice-Presidents-elect, happily designated them by titles that will stick; e. g., Cantrell, "The Tall Sycamore of Hunt," Shropshire, one of the bandwagon's wheelhorses, "The Mighty Sampson of the Southwest." Then the officers had to be again presented to the full convention when the House adjourned to the auditorium and joined the scientific body, and speeches were again called for, all of which our reporter again failed to get.

Councilors.—First District, J. T. Turner, El Paso; Second District, P. C. Coleman, Colorado; Third District, D. R. Fly, Amarillo; Fourth District, C. M. Alexander, Coleman; Fifth District, W. B. Russ, San Antonio; Sixth District, H. J. Hamilton, Laredo; Seventh District, T. J. Bennett, Austin; Eighth District, W. A. Rape, Victoria; Ninth District, H. B. Dechard, Galveston; Tenth District, B. F. Calhoun, Beaumont; Eleventh District, S. R. Burroughs, Buffalo; Twelfth District, G. B. Foscue, Waco; Thirteenth District, J. H. McCracken, Mineral Wells; Fourteenth District, Holman Taylor, Marshall.

Delegates to American Medical Association.—S. R. Burroughs, Buffalo; Alternate, J. A. Dodson, Vernon; F. Paschal, San Antonio; Alternate, T. C. Whitehead, Del Rio; J. T. Wilson, Sherman; Alternate, C. M. Rosser, Dallas; R. W. Knox, Houston; Alternate, W. J. Jameson, Palestine; J. W. McLaughlin, Galveston; Alternate, A. C. Scott, Temple.

Orator.—Dr. J. D. Law (we'll have a good oration sure), Belton.

Time and place of meeting, 1905, Houston, April 24, 25, 26 and 27.

There were over one hundred papers on the program, about one-half were read and discussed. Among them were some of exceptional value. They will all appear in the Transactions, many in the *Journal American Medical Association,* and some, from time to time, in the "Red Back."

There was so much to be done to get started that there was not time to hold any memorial services at all.

The President announced the following standing committees:

*Public Printing and Legislation.*—J. T. Wilson, Sherman; C. E. Cantrell, Greenville; T. J. Bennett, Austin; F. E. Daniel, President, ex-officio; I. C. Chase, Secretary, ex-officio.

*Scientific Work.*—I. C. Chase, Secretary, ex-officio; T. T. Jackson, San Antonio; G. B. Foscue, Waco.

*Publication.*—I. C. Chase, Secretary, ex-officio; W. R. Thompson, Fort Worth; D. R. Fly, Amarillo.

*Arrangements.*—S. C. Red, R. W. Knox, J. W. Scott, Houston.

PURE FOOD BILL.—By unanimous vote the Secretary was instructed to write to Senator Heyburn, that it is the sense and desire of the medical profession of Texas, as represented in their State organization, that the pure food bill should pass, extending to him their endorsement and support and pledging him their assistance in every way possible with members of Congress.

DISTINGUISHED GUESTS.—The Association was honored by the presence of Dr. C. A. L. Reed, of Cincinnati, ex-President American Medical Association and chairman of the Committee on Legislation. Dr. Reed met with the Association's Committee on Legislation, and a plan of co-operation was adopted. He also addressed the House of Delegates. It was much regretted that he was obliged to leave before the fun commenced; though he was the recipient, while here, of some distinguished attentions. He is a very attractive man, known all over the world.

We had with us also that elegant old Virginian gentleman, Dr. H. R. Carter, of the United States Public Health and Marine Hospital Service; the famous yellow fever expert. He favored the Association with a splendid lecture on the *Stegomyia fasciata.* Dr. Carter is from Louisa county, "Old Ferginny," and is kin to Colonel Kyarter, of Kyartersville. It is a delight to know him.

Dr. Jno. Punton, of Kansas City, was also a visitor; the well known editor of the Kansas City *Lancet Index,* and authority on nervous diseases. It was he who detected that Oran Hoskins was malinguering, and saved the railroad company $35,000, judgment for which amount had been awarded by the court at Fort Worth, Texas; and sent Oran where he ought to be.

Our old-time friend, R. H. L. Bibb, of Saltillo, Mexico, Chief Surgeon Mexican National Railroad, was also with us a short while, arriving only on the last day. He was in conference with State Health Officer Tabor and put in very little time at the meetings. He was a distinguished figure, however, at the grand ball. Bibb is a Virginian, also a Texan by adoption, and was, in 1882, Secretary State Medical Association. He expatriated himself, much to our regret, and has been living in Mexico since 1884. Bibb is a power in Mexico, and a delightful fellow everywhere. No man has more or more loyal friends.

OF THE SOCIAL FEATURES OF THE CONVENTION WEEK, I will say they were elegant and delightful, and much enjoyed and appreciated. The receptions at the Governor's, at the Drs. Wooten, and at Seton Infirmary and Mrs. Bennett's drive were attended by many of the physicians and by most of the visiting ladies. The festivities closed Friday night by a grand reception and ball and supper at the Driskill. Over one thousand sat down to supper. In the ball room the music rose with its voluptuous swell, on time, per schedule, and the soft eyes looked love, all right, and the lamps shown bright over fair women and brave men, as usual, and so forth. The Travelers' Protective Association tendered the doctors a ball also, Saturday night, but few remained to attend it.

A SECTION ON RAILROAD SURGERY was created, and a Committee on Revision and Memorials was appointed. They will revise the existing nine sections, making some changes in the name and organization, and report at next meeting.

A resolution was adopted thanking the President and Faculty of the University for numerous courtesies.

The appointment of Section officers is announced below. Delegates are elected to serve two years, and are not eligible as chairmen or secretary of Sections. Neither can the Councilors serve. It is understood that it will be the President's policy to not appoint on the Sections any member who has served either as chairman or secretary of any section or held one of the offices within recent years; but to recognize some of the younger men who have heretofore not been honored, and bring them forward and put them in line and training for future officers and leaders, succeeding the present generation; and to give every section of the State representation.

SECTION OFFICERS.

*Section on General Medicine.*—W. B. Collins, Chairman, Lovelady; S. P. Rice, Secretary, Marlin.

*Section on Obstetrics and Diseases of Women and Children.*—W. R. Blailock, Chairman, McGregor; J. C. Ervin, Secretary, McKinney.

*Section on Surgery.*—J. H. Reuss, Chairman, Cuero; I. E. Clark, Secretary, Schulenberg.

*Section on Psychology and Medical Jurisprudence.*—J. S. Turner, Chairman, Terrell; J. S. Lankford, Secretary, San Antonio.

*Section on State Medicine and Public Hygiene.*—M. M. Smith, Chairman, Austin; H. W. Cummings, Secretary, Hearne.

*Section on Gynecology.*—J. E. Gilcreest, Chairman, Gainesville; O. I. Halbert, Secretary, Waco.

*Section on Ophthalmology, Otology, Rhinology and Laryngology.*—E. H. Carey, Chairman, Dallas; J. H. Burleson, Secretary, San Antonio.

*Section on Dermatology.*—F. R. Martin, Chairman, Kyle; M. L. Moody, Secretary, Greenville.

*Section on Pathology.*—M. B. Grace, Chairman, Seguin; R. T. Morris, Secretary, Houston.

*Section on Railroad Surgery.*—C. A. Smith, Chairman, Tyler; W. G. Jameson, Secretary, Palestine.

PRESENTATION OF GAVEL.—An interesting incident was the presentation by retiring President Paschal to his successor, of a beautiful gavel, made from the wood of a tree growing on the spot where the immortals Milam, Crockett, Travis and Bowie camped the night before the battle of the Alamo. It is about a mile from the historic Alamo. The gavel is finely polished and beautifully bound and ornamented with silver. On it is a Latin inscription dedicating it to the cause of Truth, Health and Humanity, and on the front is a silver star; on the lower band is inscribed: "As I sheltered and protected the Sentinels of Texas Liberty, so will I watch over the interests of the State Medical Association of Texas." Dr. Paschal presented the gavel with a beautiful and eloquent address, and it was accepted by Dr. Daniel in appropriate words.

THE EXHIBITS were beautiful and numerous. We were much gratified. At all hours the corridors of the great auditorium, where the exhibits were installed, were thronged by doctors and citizens and college people, and a splendid sprinkle of prettily dressed women.

Mellin's Food was prominently in evidence, a fine display, presided over by the genial and immensely popular Col. Henry A. Snyder, the best known "detail man" in Texas.

E. Fougera & Co.'s beautiful display of their famous pharmaceutical specialties was in charge of the courteous Henry Copleston, a Texas man, by the bye, with whom my acquaintance began in Fort Worth away back in 1882.

The doctors flocked around the tables, and were delighted to get samples of the famous "Colchi-Sal," Cypridol, Apioline, Morrhual Creosote Capsules, and the "Clin." preparations. They had seen them advertised in the "Red Back," and it was like meeting old acquaintances.

The "Denver fellows" (Denver Chemical Co., N. Y.) were represented by a big, handsome fellow, Dr. Joseph Weiss. Not a doctor who met him will ever forget him, or the wonderful product of their laboratory—Antiphlogistine. This preparation has taken like wild fire, and the sales in Texas are immense.

The Appletons were there with a fine display of medical books. It attracted much attention.

The *Medical Recorder*, our young and sprightly neighbor at Shreveport—only four months old—but it crows lustily, was represented by a nice young lady, Miss Lucy Lane, and for one day the lordly Oscar Dowling, the handsome editor, was on hand, and charmed the Texas doctors. We are getting jealous.

The *Medical News* had a fine show-up under the management of Miss Wooldridge; and last, but not least, the famous "Red Back" was on deck, and Mrs. and Miss Hicks, who dispensed its favors and took in the cash, did a rattling business.

Then there was a fine display of surgical instruments by the Kirby Co., of Dallas; Dr. McClure, President of the Co. and Dr. Vick, being in charge. Another fine display of ditto, plus office furniture by the A. P. Cary Co., Dallas, and the Spears Co., of San Antonio.

Then there was a handsome and polite young fellow in charge of Horlick's Malted Milk, and the way the girls and "boys" (I mean the "ladies" and "doctors") did flock around that fountain flowing with malted milk and melted honey, was amazing.

Sharp & Dohme were represented by the cheery, the dear old big sunflower, the ubiquitous, Cornell, with a happy smile and a friendly grip for every one. They had no display.

The Houston Optical Co. also had a pretty lay out, in charge of the head of the firm. I can't recall any others. There was a Chicago publishing house represented by Dr. McCormack.

Oh, take it altogether, we just had a delightful time; a time to be long remembered.

---

Our Medical Licensing Board.—By reference to report herewith published it will be seen that license was refused fifty-six applicants out of ninety-three. The State of Texas has cause to be proud of the showing. It demonstrates that, although we have a defective law, one that exempts the "drugless ones" from examination (please, God, we will knock that out next Legislature), we have a good Board, and they have fixed the standard of requirements high. The examinations, while of an eminently practical kind, are rigid and thorough. Illiteracy was the cause of rejection of many. You can not make a silk purse out of a sow's ear; nor, as the lamented Love said, you can not make milk punch out of a cow's ear; nor can you make a physician out of an uneducated man. No one should be licensed to practice medicine who can not write the English language at least with a fair degree of accuracy, and a decent regard for the ordinary rules of grammar. Think of licensing a fellow to practice medicine who spells Texas with a little "t," and capitalizes "it," "may," "be," "but," etc. One of these rejected fellows wrote, "it is proBible."

Fifty-six rejections out of ninety-three applications. That is a record breaker. I hope it will deter plow boys from trying to practice medicine before they learn to read and write. Let me see; that is over 60 per cent. Good. There are too many offerings. We must skim off the cream for use and throw the blue John to the pigs. Let none but educated men try to pass the Texas Board. I am opposed to recognizing license from *any State* Board whatever. Let us do our own licensing. If a man can pass Virginia or Kentucky or Illinois, he oughn't to be afraid to try Texas.

---

To THE MEMBERS OF THE STATE MEDICAL ASSOCIATION OF TEXAS.—Now that a great interest has been awakened in organized medicine, every member who feels the interest in it that he should feel, will want to keep in touch with its work, and be informed of all reform proposed and to know the news generally, concerning our interests. To this end, every one not already a subscriber should at once enroll under the banner of the "Red Back," the champion and defender of legitimate medicine and its interests, and for twenty years a constant advocate of the highest and purest standard of professional character. Besides the Association matters, of which it will be a monthly exponent, there will always be found in its pages high-class papers by able writers, and much else that an up-to-date physician will value and appreciate. Send in your names now. If not convenient to send the $1.00, you can pay "the pretty young lady" at the next meeting.

## Figures and Tables

**Figure f1:**
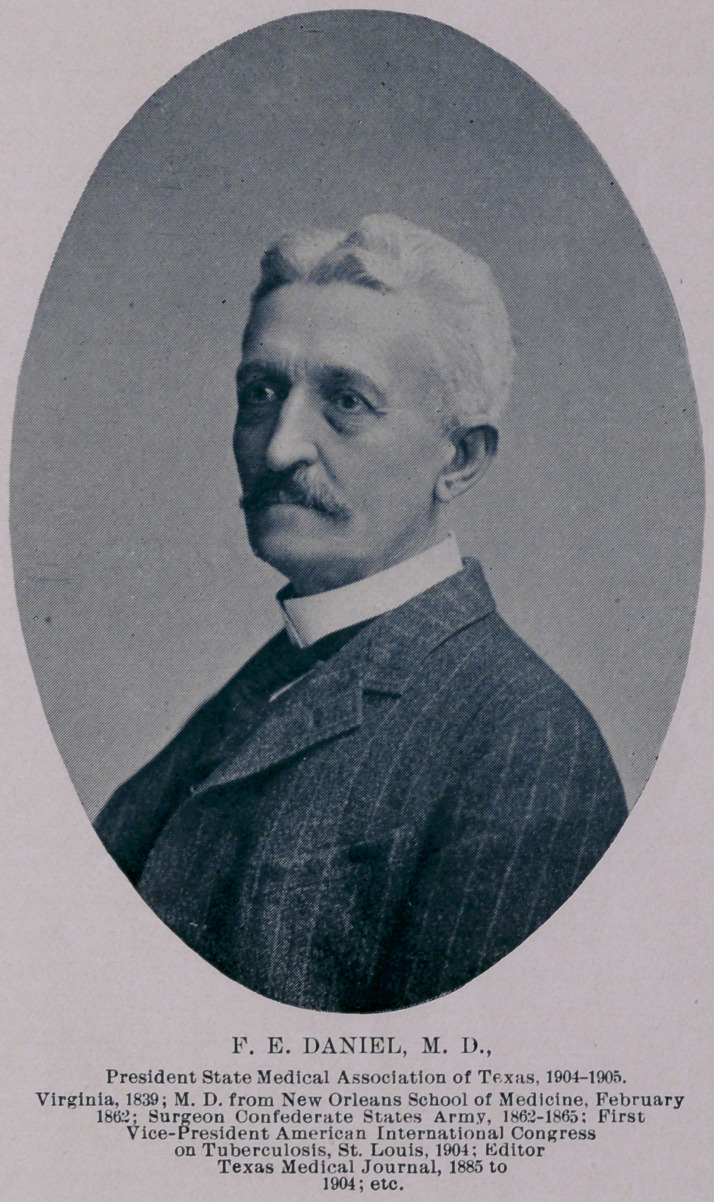


**Figure f2:**